# Prognostic Implication of Preoperative Behavior Changes in Patients with Primary High-Grade Meningiomas

**DOI:** 10.1155/2014/398295

**Published:** 2014-01-21

**Authors:** Andrej Vranic, Frederic Gilbert

**Affiliations:** ^1^Department of Neurosurgery, University Medical Centre Ljubljana (UMC Ljubljana), Zaloska 7, 1000 Ljubljana, Slovenia; ^2^Ethics & Bionics/Nanomedicine, Australian Centre of Excellence in Electromaterials Science, University of Tasmania, 7000 Hobart, Australia

## Abstract

High-grade meningiomas are rare extra-axial tumors, frequently causing brain invasion and prominent brain edema. Patients harboring high-grade meningiomas occasionally present with behavior changes. Data about frequency and prognostic importance of preoperative behavior changes in patients with high-grade meningiomas is missing. 86 patients with primary high-grade meningiomas were analyzed. Statistical analysis was performed to determine correlation of preoperative behavior changes with tumor location, preoperative brain edema, tumor cleavability, tumor grade, Ki67 proliferation index, and microscopic brain invasion. Survival analysis was performed. 30 (34.9%) patients presented with preoperative behavior changes. These changes were more frequent with male patients (*P* = 0.066) and patients older than 55 years (*P* = 0.018). They correlated with frontal location (*P* = 0.013), tumor size (*P* = 0.023), microscopic brain invasion (*P* = 0.015), and brain edema (*P* = 0.006). Preoperative behavior changes did not correlate with duration of symptoms, tumor cleavability, tumor malignancy grade, and Ki67 proliferation index. They were not significantly related to overall survival or recurrence-free survival of patients with primary high-grade meningiomas. Preoperative behavior changes are frequent in patients harboring primary high-grade meningiomas. They correlate with tumor size, microscopic brain invasion, and brain edema. Preoperative behavior changes do not predict prognosis in patients with primary high-grade meningiomas.

## 1. Introduction

Meningiomas represent the most frequently diagnosed primary central nervous system tumors in adults over 35 years, accounting for 33.8% in the USA between 2004 and 2006 [1]. Women are affected twice as commonly as men. According to the WHO classification of brain tumors [2], about 3% of meningiomas are malignant (WHO Grade III) and about 20% are atypical (WHO Grade II). The rest account for benign meningiomas (WHO Grade I). 

While benign meningiomas in general are encapsulated and slowly growing, high-grade (Grade II/III) meningiomas often recur. Recurrence rates up to 80% for Grade II and up to 100% for Grade III meningiomas in 20 years have been reported [3]. Tumor grade, microscopical brain invasion, high mitotic count, and parasagittal location are negative prognostic factors of recurrence-free survival of patients with high-grade meningiomas [3, 4].

Most frequent presenting signs of high-grade meningioma patients are epileptic seizures, hemiparesis, and headache [5]. Mean duration of symptoms is 7.5 months prior to diagnosis [6]. There is no difference in duration of symptoms between primary and recurrent high-grade meningiomas [7]. So far, no type of presenting symptoms has been confirmed as a prognostic sign for meningioma recurrence. Both low- and high-grade meningioma patients may also present with behavior changes, frequently described as psychoorganic syndrome. Data about frequency and prognostic importance of preoperative behavior changes in patients with brain tumors, including patients with high-grade meningiomas, is missing [8].

In the present study, we analyzed 86 consecutive patients with primary high-grade meningiomas, treated at a single institution. Our aim was to assess the frequency, correlation with other factors, and prognostic importance of preoperative behavior changes in patients with primary high-grade meningiomas, which, to the best of our knowledge, have so far not been evaluated yet.

## 2. Materials and Methods

### 2.1. Patients

86 consecutive patients with primary high-grade meningiomas diagnosed between 1990 and 2005 were included in the study. In the same time period, 790 primary benign meningiomas were diagnosed. All patients were surgically treated at the Department of Neurosurgery, University Medical Centre, Ljubljana, Slovenia. Selection of patients was based on original pathological reports. All original histological slides were reviewed when the original diagnosis was either Grade II or Grade III meningioma. Cases were reviewed without knowledge of clinical outcome and other clinical data. Microscopical brain invasion was assessed separately, and Ki67 proliferative index, which is a measure of cellular proliferation, was determined using routine immunohistochemical protocols.

Clinical data were retrieved from the patients' medical records. The following clinical data were collected: tumor site (L/R), location and size, preoperative signs and symptoms, duration of symptoms, presence of brain edema on preoperative MR scans, tumor cleavability, and extent of surgical resection. Brain edema was graded as absent, mild, or severe. Data on cleavability were obtained from original operative reports with special emphasis on the tumor/brain interface. Meningiomas were classified as noncleavable when pial invasion was observed by the surgeon and cleavable when dissection in the extrapial plane could be performed [9].

Data on preoperative behavior changes were also retrieved from patients' medical records. Patients were classified as having preoperative behavior changes if any data about behavior changes was noted in their history.

Follow-up data were collected for all patients, including recurrence-free survival (RFS; time from surgical resection to local recurrence) and overall survival (OS; time from surgical resection to death). The dates of patients' death were collected from the Cancer Registry of Slovenia. No attempts were made to determine the cause of death.

### 2.2. Statistical Analysis

Statistical analysis was performed with SPSS 16.0 for Windows (SPSS Inc., Chicago, IL). The differences between groups in relation to other clinicopathological parameters were analyzed by the Mann-Whitney and the Kruskal-Wallis tests. Survival analysis was performed by the Cox model and survival curves were estimated using the Kaplan-Meier method. The final multivariate model was achieved using a stepwise backward elimination of nonsignificant variables.

## 3. Results

86 consecutive patients with primary high-grade meningiomas were analyzed. Among 86 high-grade meningiomas cases, 76 meningiomas (88%) were Grade II and 10 (12%) were Grade III. 42 patients were male (49%) and 44 (51%) were female.

Out of 86 patients analyzed, clinical data was available for 83 patients. Behavior of 30 patients (34.9%) was described by their relatives as being changed. Out of these 30 patients, behavior changes were a *presenting symptom* in 12 cases (14%). Other patients presented with headaches (23 patients; 26.7%), hemiparesis (22 patients; 25.6%), epileptic fits (20 patients; 23.3%), and cranial nerves palsy (6 patients; 7%). In the group with behavior changes, patients were mostly suffering from aggressiveness, although other changes like apathy or hypersexuality were also described. Clinical and pathological characteristics of 86 high-grade meningiomas are shown in Table 1.

Patients with behavior changes were older (mean 57.1 years) than patients with normal preoperative behavior (mean 53.7 years) (*P* = 0.017). Behavior changes tended to be more frequent in male patients: they were present in 19 out of 42 (47.5%) male and 11 out of 44 (27.5%) female patients (*P* = 0.066). They were found more frequently in larger tumors (*P* = 0.023) and were strongly associated with presence of brain edema (*P* = 0.006). Preoperative behavior changes were present in 31 (53.4%) cases with mild edema and in 17 (29.3%) cases with severe brain edema, while absent in all cases with no brain edema. They were also strongly correlated to tumor location, being more frequent in patients with tumors located frontally or frontobasally (*P* = 0.013). Preoperative behavior changes were less frequent in tumors located parasagittally (*P* = 0.008) and were not correlated to the site (L/R) of the tumor (*P* = 0.298). While there was a strong correlation of behavior changes with microscopic brain invasion (*P* = 0.015), no correlation with tumor cleavability as assessed by the surgeon was noticed. There was also no correlation between preoperative behavior changes and duration of symptoms, WHO Grade of the tumor, or Ki67 proliferation index. Correlation of several factors with preoperative behavior changes is shown in Table 2.

Patients were followed until death or for a median of 96 months (range from 28 to 204 months). The 5- and 10-year recurrence-free survival (RFS) for the entire group of 86 high-grade meningiomas was 60% (95% CI, 48–71) and 55% (95% CI, 43–68), respectively. The 5- and 10-year overall survival (OS) for the entire group was 76% (95% CI, 67–86) and 56% (95% CI, 44–68), respectively. The univariate Cox analyses of the RFS and OS are shown in Table 3. On multivariate analysis, brain invasion and parasagittal-falcine location were independent negative predictors of RFS, while male gender and parasagittal-falcine location were the only parameters associated with decreased OS. There were no significant differences in RFS or OS between patients with or without preoperative behavior changes.

## 4. Discussion

Psychoorganic syndrome is a vague definition of behavior changes caused by a morphologically proven lesion in the brain. It has been described in patients with brain tumors, including meningiomas [10–13], revealing a dilemma whether neuroradiologic imaging should be a part of the routine diagnostic procedure in all psychiatric patients.

Most frequent behavior changes in patients with brain tumors are slowness, apathy, aggressiveness, hypersexuality, social inappropriateness, poor judgment, and lack of planning and rarely hallucinations and other psychotic symptoms [11]. Preoperative behavior changes are frequent findings in patients with *butterfly gliomas* (i.e., *glioblastoma multiforme* affecting both frontal lobes) or benign *olfactory meningiomas *[10, 11]. These two conditions affect both frontal lobes, causing malfunction of the prefrontal cortex. Several cases of unilateral frontal or frontobasal tumors causing behavior changes have been described [13–15]. Correlation with frontal or frontobasal location was also confirmed in our study, even if the tumor was unilateral. However, preoperative behavior changes are not limited to patients with frontal lobe tumors. Rare cases of unusual acquired aggressive and antisocial behavior correlated with frontal, temporal, thalamic, hypothalamic, or left parietal tumors have been reported in the literature [16–22]. So far, reviews have demonstrated no correlation between specific psychiatric symptoms and tumor location or histological type [23]. Since it was impossible to specify the behavior changes, no correlation between the type of behavior changes and the location of the tumor could be performed in our retrospective study.

We have shown that preoperative behavior changes, although not prognostically important, present a frequent finding in patients with high-grade meningiomas. According to our results, preoperative behavior changes mostly correlate with tumor invasion in the surrounding brain, as well as with brain edema. In high-grade meningioma patients, brain edema is more the result of tumor invasion than venous compression. Behavior changes do not correlate with other tumor malignancy properties, such as cellular proliferation, measured with the Ki67 proliferation index, neither do they correlate with the meningioma grade. Therefore, behavior changes may be the result of tumor cell invasion into the brain parenchyma as well as of brain edema forming in the invaded brain. Less probably, they may develop as the result of direct pressure on the brain parenchyma by a rapidly proliferating tumor.

Behavior changes may be the only initial manifestation of meningiomas [8]. Our study confirmed that in 14% they are a presenting symptom in patients with high-grade meningiomas. Since growth rate of high-grade meningiomas is relatively slow compared to malignant gliomas, the onset of behavior changes is slow, behavior changes are subtle, and patients with high-grade meningiomas may first be referred to psychiatrists [24]. In these cases, if radiological imaging is not performed, the accurate diagnosis may only emerge when the tumor has grown large enough to compress the brainstem and disturb other brain functions, such as consciousness [25].

A recent study reported significant risk for brain tumors in the first 1-2 years after admittance to a psychiatric hospital [26]. Nevertheless, studies have reported no raised incidence of intracranial tumors among patients manifesting schizophrenia, affective illness, or neurotic symptoms [27]. A very small incidence of positive radiological findings in psychiatric patients, except for brain atrophy, does not give good reason for routine CT and MRI brain scans in all hospitalized psychiatric patients [28].

In our study, we have showed that patients with high-grade meningiomas frequently manifest behavior changes, sometimes even as presenting symptoms. Since prognosis in these patients is worse than prognosis of patients with benign meningiomas—time to recurrence in malignant meningiomas is less than one year and no chemotherapy is effective—it is important not to delay the diagnosis. Early diagnosis gives the best chance of successful treatment to high-grade meningioma patients. The possibility of a patient suffering from behavior changes due to high-grade meningioma, initially being admitted to a psychiatric facility, illustrates how initial evaluation is critical. In these rare cases, transition from psychiatric to neurosurgical care management should be accelerated and surgical treatment should be performed immediately. In the process of diagnostics of patients with subtle behavior changes, the idea of high-grade meningioma must not be neglected.

Methodological shortcoming of this study is especially the retrospective nature of the study, without the use of a structured questionnaire to determine the nature of behavior changes. Correlation between the type of behavior changes and tumor location would add a lot to understanding the nature of psychiatric symptoms in patients with brain tumors.

## 5. Conclusions

Preoperative behavior changes are frequent in patients harboring primary high-grade meningiomas. They correlate with tumor size, microscopic brain invasion, and brain edema. Preoperative behavior changes do not predict prognosis in patients with primary high-grade meningiomas.

## Figures and Tables

**Table 1:** Clinical characteristics of 86 cases of high-grade meningiomas.

Parameter	n* (%)
Gender	86
Male	42 (48.8)
Female	44 (51.2)

Age	86
Range, 13.5–85.4 years	—
Mean, 55.0 years	—
Median, 57.2 years	—

Tumor size	78
Range, 2.0–8.5 cm	—
Mean, 4.9 cm	—
Median, 5.0 cm	—

Tumor location	86
Parasagittal-falcine	26 (30.2)
Convexity	35 (40.7)
Base	24 (27.9)
Frontal/frontobasal	16 (18.6)

Tumor site	86
Left	37
Right	31
Bilateral	18

Duration of signs/symptoms	80
Range, 0–36 months	—
Mean, 7.5 months	—
Median, 4.0 months	—

Presenting signs/symptoms	83
Headache	23 (25.9)
Hemiparesis	22 (36.6)
Seizures	20 (24.7)
Behavior changes	12 (14)
Cranial nerve palsy	6 (11.1)

Behavior changes	83
Present	30 (36.1)
Absent	53 (73.9)

Edema	58
Absent	10 (17.2)
Mild	31 (53.4)
Severe	17 (29.3)

Cleavability	42
Cleavable	18 (42.9)
Noncleavable	24 (57.1)

*The information on some parameters was not available for all patients.

**Table 2:** Correlation of several factors with preoperative behavior changes.

Brain edema	0.006
Nonparasagittal location	0.008
Frontal/frontobasal location	0.013
Brain invasion	0.015
Age	0.018
Tumor size	0.023
Sex	0.066
Cleavability	0.233
Tumor site L/R	0.298
Ki67	0.404
Duration of symptoms	0.626
WHO Grade	0.650

**Table 3:** Univariate survival analysis of 86 high-grade meningiomas.

Variable	*n*	Recurrence-free survival			Overall survival		
		HR	95% CI	*P* value	HR	95% CI	*P* value
Sex (male versus female)	86	1.9	0.9–4.0	0.077	1.9	1.0–3.8	0.064
Age (≥55 versus <55)	86	0.9	0.5–1.8	0.776	3.19	1.5–6.7	0.002
Location (parasagittal-falcine versus others)	86	2.3	1.1–4.7	0.021	1.5	0.8–3.0	0.238
WHO Grade (III versus II)	86	2.0	0.8–5.3	0.145	1.7	0.6–4.3	0.293
Brain invasion (present versus absent)	37	7.2	0.9–55.7	0.059	4.4	1.0–19.2	0.049
Ki67 (>4% versus ≤4%)	86	2.7	1.1–6.7	0.027	1.9	0.9–4.0	0.104
Behavior changes (present versus absent)	83	0.7	0.3–1.7	0.514	1.0	0.5–2.0	0.112

## References

[1] Kruchko C (2010). *Central Brain Tumor Registry of the United States*.

[2] Perry A, Louis DN, Scheithauer BW, Louis DN, Ohgaki H, Wiestler OD (2007). . Meningiomas. *World Health Organisation Classification of Tumours: WHO Classification of Tumours of the Central Nervous System*.

[3] Jaaskelainen J, Haltia M, Laasonen E (1985). The growth rate of intracranial meningiomas and its relation to histology. An analysis of 43 patients. *Surgical Neurology*.

[4] Durand A, Labrousse F, Jouvet A (2009). WHO grade II and III meningiomas: a study of prognostic factors. *Journal of Neuro-Oncology*.

[5] Mahmood A, Caccamo DV, Tomecek FJ, Malik GM, Black PM, Kepes JJ (1993). Atypical and malignant meningiomas: a clinicopathological review. *Neurosurgery*.

[6] Vranic A, Popovic M, Cör A, Prestor B, Pizem J (2010). Mitotic count, brain invasion, and location are independent predictors of recurrence-free survival in primary atypical and malignant meningiomas: a study of 86 patients. *Neurosurgery*.

[7] Ayerbe J, Lobato DR, De la Cruz J (1999). Risk factors predicting recurrence in patients operated on for intracranial meningioma. A multivariate analysis. *Acta Neurochirurgica*.

[8] Gupta RK, Kumar R (2004). Benign brain tumours and psychiatric morbidity: a 5-years retrospective data analysis. *Australian and New Zealand Journal of Psychiatry*.

[9] Alvernia JE, Sindou MP (2004). Preoperative neuroimaging findings as a predictor of the surgical plane of cleavage: prospective study of 100 consecutive cases of intracranial meningioma. *Journal of Neurosurgery*.

[10] Gilbert F, Vranic A, Hurst S (2013). Involuntary & voluntary invasive brain surgery: ethical issues related to acquired aggressiveness. *Neuroethics*.

[11] Barrash J, Tranel D, Anderson SW (2000). Acquired personality disturbances associated with bilateral damage to the ventromedial prefrontal region. *Developmental Neuropsychology*.

[12] Dae Kyu Lee DKL, Dong Gyu Kim DGK, Choe G, Chi JG, Jung H-W (2001). Chordoid meningioma with polyclonal gammopathy. Case report. *Journal of Neurosurgery*.

[13] Avery TL (1971). Seven cases of frontal tumour with psychiatric presentation. *British Journal of Psychiatry*.

[14] Hunter R, Blackwood W, Bull J (1968). Three cases of frontal meningiomas presenting psychiatrically. *British Medical Journal*.

[15] Burns JM, Swerdlow RH (2003). Right orbitofrontal tumor with pedophilia symptom and constructional apraxia sign. *Archives of Neurology*.

[16] Miller BL, Cummings JL, McIntyre H (1986). Hypersexuality or altered sexual preference following brain injury. *Journal of Neurology Neurosurgery and Psychiatry*.

[17] Caplan R, Comair Y, Shewmon DA, Jackson L, Chugani HT, Peacock WJ (1992). Intractable seizures, compulsions, and coprolalia: a pediatric case study. *Journal of Neuropsychiatry and Clinical Neurosciences*.

[18] Villano LJ, Mlinarevich N, Watson KS, Engelhard HH, Anderson-Shaw L (2009). Aggression in a patient with primary brain tumor: ethical implications for best management. *Journal of Neuro-Oncology*.

[19] Haugh RM, Markesbery WR (1983). Hypothalamic astrocytoma. Syndrome of hyperphagia, obesity, and disturbances of behavior and endocrine and autonomic function. *Archives of Neurology*.

[20] Nakaji P, Meltzer HS, Singel SA, Alksne JF (2003). Improvement of aggressive and antisocial behavior after resection of temporal lobe tumors. *Pediatrics*.

[21] Kaplan PW, Kerr DA, Olivi A (1999). Ictus expectoratus: a sign of complex partial seizures usually of non-dominant temporal lobe origin. *Seizure*.

[22] Partlow GD, Carpio-O’Donovan R, Melanson D, Peters TM (1992). Bilateral thalamic glioma: review of eight cases with personality change and mental deterioration. *American Journal of Neuroradiology*.

[23] Madhusoodanan S, Danan D, Moise D (2007). Psychiatric manifestations of brain tumors: diagnostic implications. *Expert Review of Neurotherapeutics*.

[24] Dumas-Duport C (1970). *Tumeurs cerebrales chez les malades mentaux [Ph.D. thesis]*.

[25] Maurice-Williams RS, Dunwoody G (1988). Late diagnosis of frontal meningiomas presenting with psychiatric symptoms. *British Medical Journal*.

[26] Benros ME, Laursen TM, Dalton SO, Mortensen PB (2009). Psychiatric disorder as a first manifestation of cancer: a 10-year population-based study. *International Journal of Cancer*.

[27] Ron MA (1989). Psychiatric manifestations of frontal lobe tumours. *British Journal of Psychiatry*.

[28] Hrabric K, Jukic V, Bilic P (2009). Neuroradiologic diagnostics in patients hospitalized in Vrapce Psychiatric Hospital. *Lijec Vjesn*.

